# Patient‐led research and Advocacy Efforts

**DOI:** 10.1002/cnr2.1657

**Published:** 2022-06-15

**Authors:** Argerie Tsimicalis, Ramandeep Singh Arora, Poonam Bagai, Neil Ranasinghe, Marcela Zubieta

**Affiliations:** ^1^ Ingram School of Nursing and Gerald Bronfman Department of Oncology Faculty of Medicine and Health Sciences, McGill University Quebec Canada; ^2^ Department of Pediatric Oncology Max Super Specialty Hospital Saket New Delhi India; ^3^ Department of Medical Projects and Social Support Program Cankids Kidscan New Delhi India; ^4^ SIOP GLobal Health Network, International Society of Paediatric Oncology SIOP Global Health Network London UK; ^5^ Fundación Nuestros Hijos (FNH) Santiago Chile

The treatment of children with cancer involves thinking transdisciplinary and building global partnerships. Many children with cancer have been cured using these methods, at least among the majority who reside in high‐income countries. In spite of this, much work remains to be done in low‐ and middle‐income countries. As Rafael et al.^1^ note, the fight continues, requiring strong partnerships and advocacy across multiple sectors. Efforts to cure children are grossly curtailed where pathways to diagnosis, access to clinical trials, and adherence to treatment remain significantly low. Across the globe, clinicians, academic researchers, and advocacy groups work together to support children with cancer. WHO's Global Initiative for Childhood Cancer launched in 2018 has given significant impetus to addressing inequities, improving access, advancing quality, and saving lives. Bringing together partners across sectors and globally to improve health and well‐being of children with cancer and their families is one of the goals; ensuring every child with cancer has the best chance to survive, to live full and abundant lives, and to live and die without suffering.

In the past, families of children with cancer have also played an important role in advancing cancer treatment, contributing to research, mobilizing resources, establishing non‐for‐profits, and advocating for local and system level transformational changes. Yet, these monumental strides and historical accolades that address significant gaps and inequalities, implement novel solutions, and memorialize the precious lives lost, rarely appear in the academic journals. This special issue of “Patient‐led Research and Advocacy Efforts,” contains 12 articles^1^, ^2^, ^3^, ^4^, ^5^, ^6^, ^7^, ^8^, ^9^, ^10^, ^11^, ^12^ by patient advocacy groups, parent and survivor organizations from Australia, Chile, Ecuador, India, New Zealand, and the United Kingdom. These articles showcase perspectives, methods, and empirical evidence for notable changes when strong partnerships are built across the cancer treatment trajectory beginning with diagnosis. In addition, this special issue emphasizes the crucial role children, and their families play in helping to co‐produce and mobilize knowledge to deal with the grim reality of childhood cancer. Without significant changes, the majority of children will continue to die and experience significant strains on their families, resulting in lost potential, reduced equality, and economic hardship.^13^

By illuminating the growing influence of patient representatives and patient groups on research methods, advocacy, and policy, we hope to encourage the childhood cancer community to further explore, partner and foster that influence. In most cases, patients and their caregivers are better at understanding their disease and lifestyle needs than most medical professionals. They also have important ideas about what research would be most beneficial to their lives, special to improve their daily quality of life. They are in the perfect position to identify research questions, offer novel solutions, take action, and advocate for knowledge mobilization. When it comes to involvement in research, Patient Advocacy Groups (PAGs) along with a parent and survivor support groups are increasingly vital (Figure 2). PAGs focus increasingly on driving quality care and achieving patient‐centricity.

A cancer diagnosis, followed by the initiation of treatment, can pose many challenges for the child and family, and they may experience feelings of shock, grief, and many unknowns. The abandonment of a child's treatment may be due to multiple factors at this time. Efforts are underway in India to introduce multimodal interventions to dramatically reduce treatment abandonment. In the absence of an official tracking system, Arora et al.^2^ describes the use and acceptability of a custom‐made tool in the form of the “You are not alone” book to be used by parent group members as patient navigators to track each child's progress in the initial stages of their treatment. A key component of implementation was the partnership with CanKids; a non‐for‐profit organization that provides significant social support^2^; Figure 1). For their approach, missing a scheduled hospital appointment was not the trigger for contacting families, but weekly contact is essential from the start of treatment.^2^ The weekly contact may be a promising intervention in our collective efforts to reduce the treatment abandonment of childhood cancer.^14^

**FIGURE 1:**
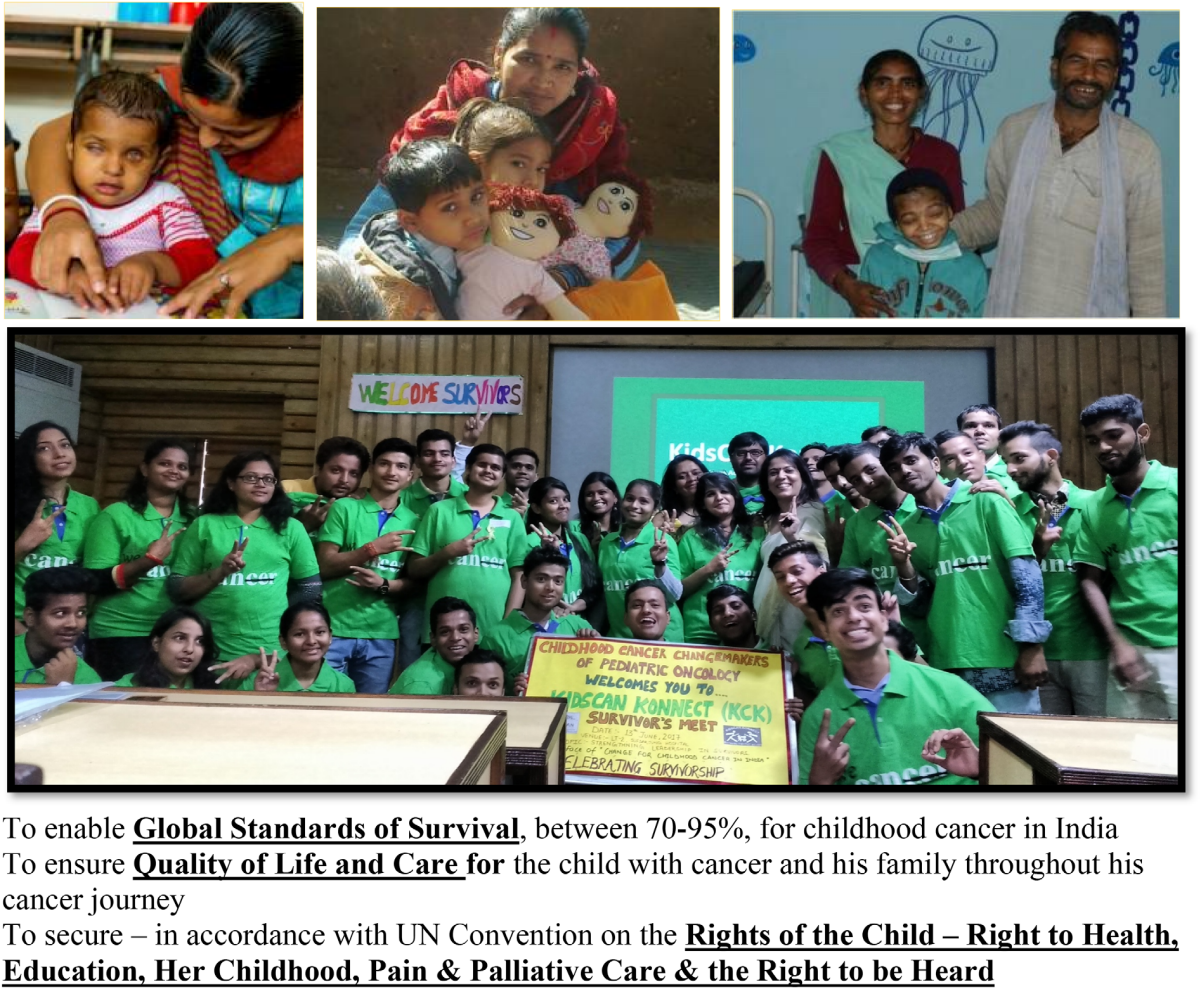
CanKids VISION is the IMPACT it seeks to make. To enable *Global Standards of Survival*, between 70% and 95%, for childhood cancer in India. To ensure *Quality of Life and Care* for the child with cancer and his family throughout his cancer journey. To secure—in accordance with UN Convention on the *Rights of the Child—Right to Health, Education, Her Childhood, Pain and Palliative Care and the Right to be Heard*

In the aftermath of the passing of Leila Rose at 22‐months, her parents, Dr Andrew and Tracy Chow, wondered how many other families suffer due to the loss of a child. Guided by the philosophy of “leaving no stone unturned,” Leila's parents established the Leila Rose Foundation in Australia.^6^ As part of the Family Support Coordinator program, the Family Support Coordinator accompanies families to medical appointments and acts as a facilitator and medical jargon interpreter.^6^ The Leila Rose Foundation also established a program designed to reduce the financial burden faced by families with a child with cancer. This program provided financial assistance for day‐to‐day bills, incidental expenses, and a limited number of whole genome analyses, as determined by the treating oncologist. Throughout the world, families are facing significant, catastrophic, and toxic financial pressures, which will affect whether they continue or abandon their child's treatment; investment and participation in research is critical (Figure 2).

**FIGURE 2:**
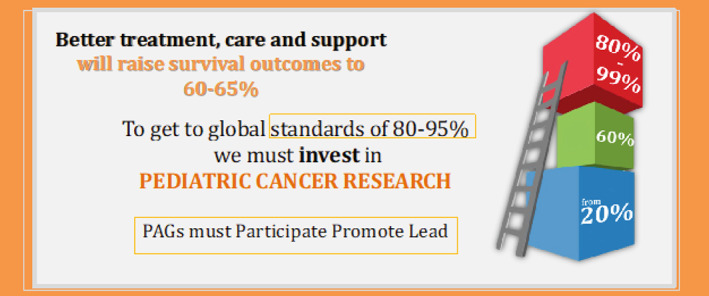
A call from CanKids for patient advocacy groups to participate, promote and lead efforts in pediatric cancer research to raise survival

As a child undergoes cancer treatment, their families will face a wide variety of needs. In order to create a support group in India, Rao et al.^11^ conducted a survey to identify the unmet needs of parents of children with cancer. Unmet needs were reported in relation to emotional concerns, informational needs, and treatment side effects. One notable unmet need was related to the provision of play/art‐based activities, and academic support for their child. As a result of a cancer diagnosis, attendance at school is disrupted, and in some cases, results in poor academic performance. Daruvala et al.^7^ describe how to set up an activity group and a learning center in Bangalore, India, with the support of a non‐governmental organization called Samiksha Foundation. Establishing these partnerships with the foundation, and mobilizing the teachers and volunteers were crucial to supporting the children. Having no empirical evidence about the timing of the return to education for children with acute lymphoblastic leukemia (ALL), Ballantine et al.^3^ sought to determine the safe time to return to social activities for children undergoing ALL treatment, and to understand how their parents perceive and react to advice related to infection risk during their child's “return to normal.” Many factors need to be taken into account when deciding when a child should return to school or social activities. The study produced five major recommendations and led to the establishment of a National Child Cancer Network working group in New Zealand. Members of the network include medical, educational, NGO, and family representatives.

As a result of the COVID‐19 pandemic, children with cancer were suddenly without their treatment facility, and their families faced financial difficulties and uncertainties. Berliner et al.^4^ report on the support activities and interventions of Fundación Nuestros Hijos (Foundation Our Children) to help Chilean families during the pandemic crisis. Children's cancer treatments were at risk during this crisis, giving rise to the slogan “Cancer does not wait.” The Fundación Nuestros Hijos delivered aid aimed at providing access to treatment, rehabilitation, and schooling. By collaborating closely, Kumar et al.^9^ describe how CanKids in India upscaled their shared care model to connect COVID designated hospitals with other hospitals to continue the treatment of children with cancer. Lastly, Celedon et al.^5^ described the implementation of a synchronous telerehabilitation program for children with cancer in Chile. Together, these authors offer insight into the barriers, facilitators, and innovative solutions to improve holistic access to care, and reduce risk of abandonment for these children with cancer during a period of unprecedented crisis.

Children and families will face a multitude of unmet needs, disruptions, and unanticipated crises when they are diagnosed with cancer, but the harsh reality is that not all children will survive. Cancer survival varies significantly by diagnosis, healthcare system, and country. In Fernández et al.,^8^ they describe their experience creating a public‐private collaboration in Chile to improve the quality of life and the end‐of‐life experience for children, adolescents, and their families. Partnerships with Fundación Nuestros Hijos have led to the creation of a palliative care program whose services are not covered by public services. However, survivors of childhood cancer lack access to healthcare services. The study by Rossell et al.^12^ highlights the importance of leveraging non‐government organizations and childhood cancer foundations in Latin America. As the children transition from cancer treatment to survivor surveillance, adult healthcare, and life beyond treatment in Latin America, this involvement can offer strategies to minimize the abrupt changes and disruptions in their care.

Polanco et al.^10^ provide a pathway for setting international standards for patient and parent participation in cancer research in children, adolescents, and young adults. Over 50 participants attended the workshop from Europe, including oncologists, nurses, allied health professionals, scientists, representatives from charity organizations, parents, and survivors. There was unanimous support for a standardized and collaborative European strategy for patient and parent involvement in pediatric oncology research. There are well‐established and long‐standing models in the UK, The Netherlands, and France, but there is considerable disparity in survival rates across Europe. In order to bridge this gap, resources must be available to raise awareness, cultivate, and foster patient and parent engagement and involvement with research. In their article Rafael et al.^1^ describe the historical contributions of the Association of Parents of Children and Adolescents with Cancer (APNACC) on the role of parents in the cancer community. An open dialog was created with the government by this passionate stakeholder in order to ensure that all types of catastrophic, rare, or orphan diseases, including cancer, are treated, free of charge in Ecuador. However, despite the fact that much remains to be done, Rafael et al.^1^ described how they created strong alliances, lobbied for changes, and secured legal rights that have saved the lives of many children with cancer.

The articles published in this issue demonstrate how patients, carers, and advocacy groups not only contribute to the improvement of cancer care for children across the cancer continuum, but also bring scientific rigor to their work and provide examples for others to follow. Furthermore, the work emphasizes the importance of patient involvement as an essential component of healthcare research. The inclusion of patient groups in fundamental and clinical research as equal partners plays a vital role in research and knowledge mobilization on the ground. The purpose of our special issue is to demonstrate how patient‐led research and advocacy can contribute to improving childhood cancer outcomes through multi‐sectoral collaboration. Our special issue illustrates innovative ways to work together, break down barriers, and dispel power dynamics in order to ***CureAll*.** ^13^

The report is intended to result in increased patient and family engagement, as well as patient organizations publishing their research and collaborating more with scientists, researchers and health care professionals in order to do so. The future looks bright (Figures 3 and 4).

**FIGURE 3:**
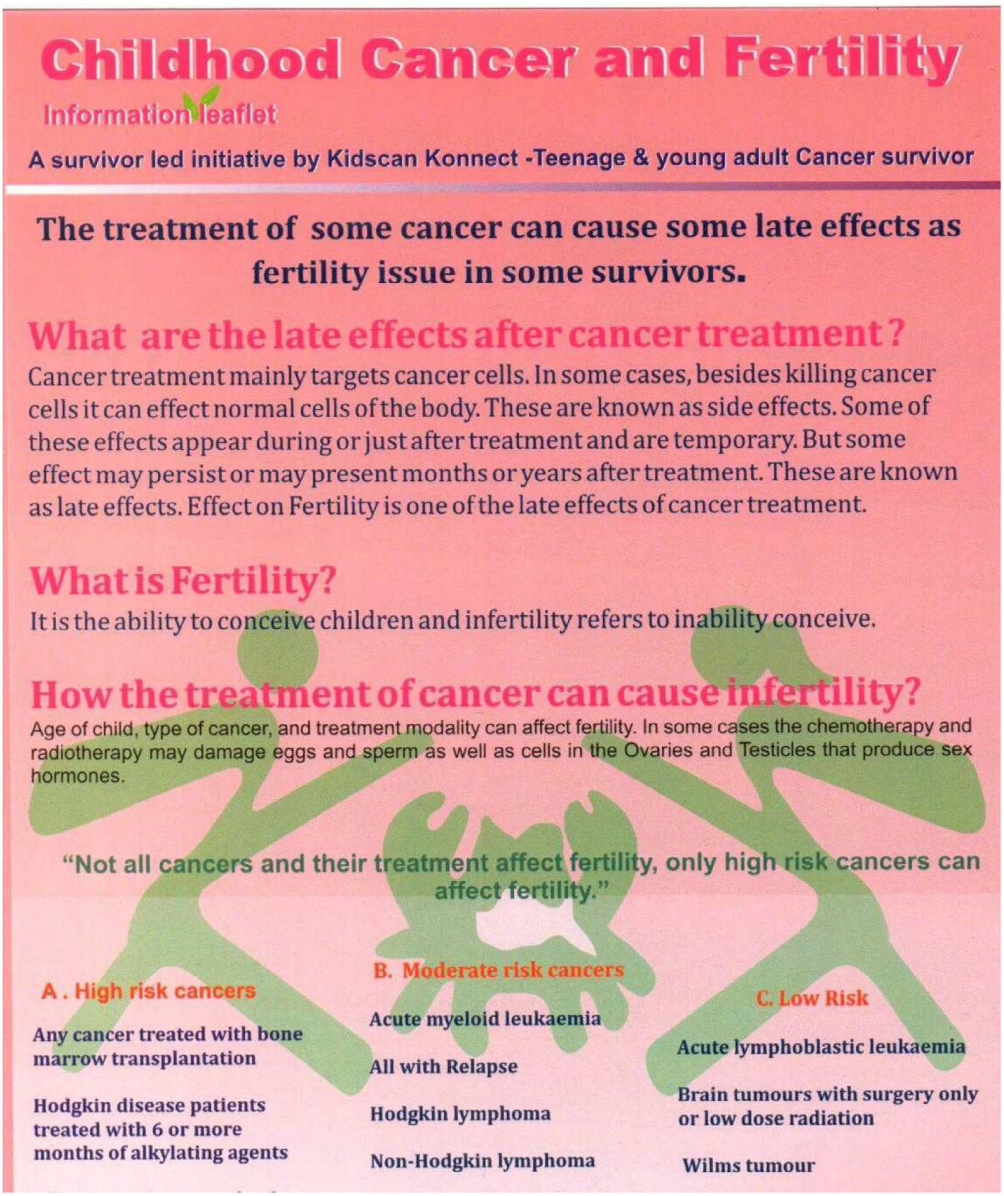
Example of patient led initiative to address late effects fertility

**FIGURE 4:**
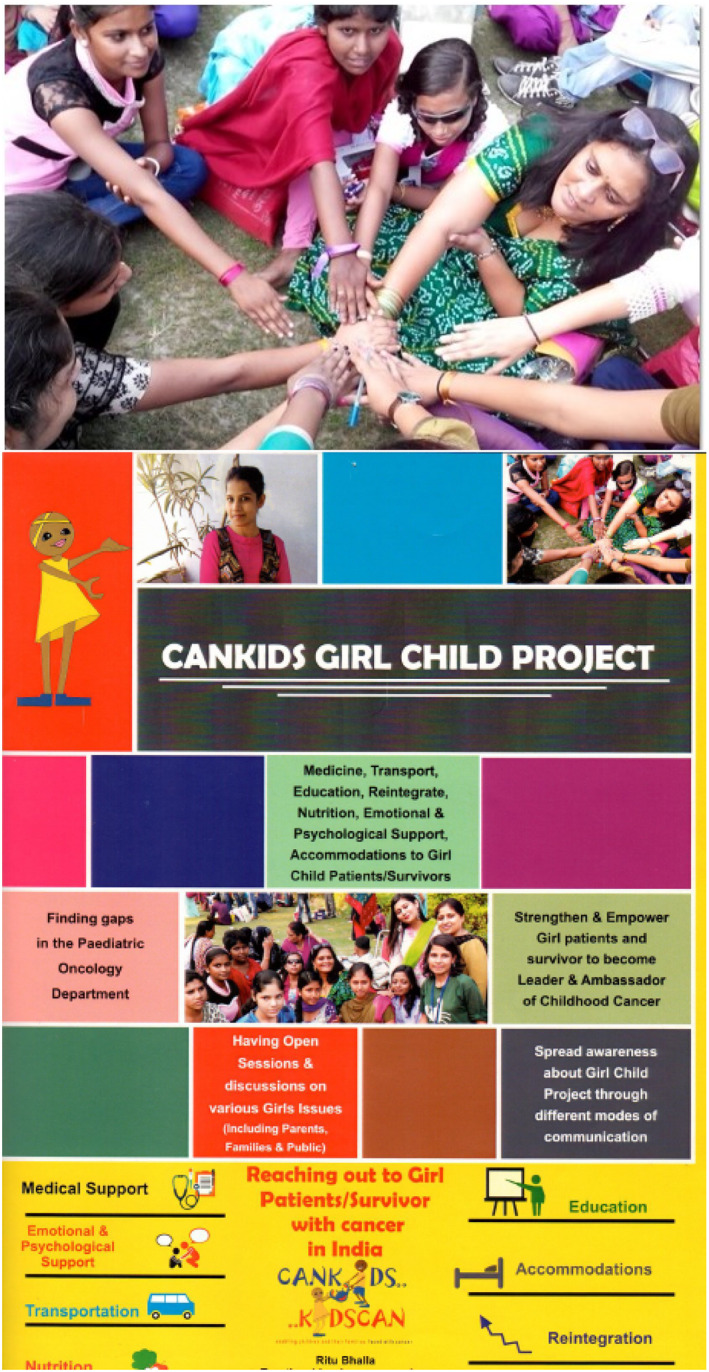
Reaching Out to Girl Patients/Survivors with Cancer in India; a CanKids Girl Child Project

## AUTHOR CONTRIBUTION

Conceptualization, A.T.; R.A.; P.B., N.R., M.Z. Writing ‐ Original Draft, A.T.; Writing ‐ Review & Editing: A.T.; R.A.; P.B., N.R., M.Z.

## CONFLICT OF INTEREST

We all declare that we have no conflicts of interest.

## ETHICS STATEMENT

This editorial did not involve human participants; hence, the editorial was not subject to an ethical review and approval process.

## Data Availability

Our editorial does not contain any research data for sharing. For questions related to our editorial piece, readers are most welcome to contact us.
